# Rheumatology clinicians’ experiences of brief training and implementation of skills to support patient self-management

**DOI:** 10.1186/1471-2474-15-108

**Published:** 2014-03-28

**Authors:** Emma Dures, Sarah Hewlett, Nicholas Ambler, Remona Jenkins, Joyce Clarke, Rachael Gooberman-Hill

**Affiliations:** 1 University of the West of England, Bristol, UK; 2 North Bristol NHS Trust, Bristol, UK; 3 University Hospitals Bristol, Bristol, UK; 4 University of Bristol, Bristol, UK

**Keywords:** Behaviour change, Cognitive-behavioural approaches, Rheumatology clinicians, Self-efficacy, Self-management, Skills training, Qualitative

## Abstract

**Background:**

Self-management of arthritis requires informed, activated patients to manage its physical and psychosocial consequences. Patient activation and self-management can be enhanced through the use of cognitive-behavioural approaches, which have a strong evidence base and provide insight into the variation in outcome of patients with ostensibly the same degree of disease activity. However, training for rheumatology health professionals in theory and skills underpinning the facilitation of self-management is not widely available. To develop such training, this study explored rheumatology clinicians’ experiences of a variety of brief skills training courses to understand which aspects were helpful or unhelpful, and to identify the barriers and facilitators of applying the skills in clinical practice.

**Methods:**

16 clinicians who had previously attended communication and self-management skills training participated in semi-structured interviews: 3 physicians, 3 physiotherapists, 4 nurses, 6 occupational therapists. Transcripts were analysed (ED) using a hybrid inductive and deductive thematic approach, with a subset independently analysed (SH, RG-H, RJ).

**Results:**

3 overarching themes captured views about training undertaken and subsequent use of approaches to facilitate self-management. In ‘putting theory into practice’, clinicians felt that generic training was not as relevant as rheumatology-specific training. They wanted a balance between theory and skills practice, and identified the importance of access to ongoing support. In ‘challenging professional identity’, models of care and working cultures influenced learning and implementation. Training often challenged a tendency to problem-solve on behalf of patients and broadened clinicians’ remit from a primary focus on physical symptoms to the mind and body interaction. In ‘enhanced practice’, clinicians viewed consultations as enhanced after training. Focus had shifted from clinicians’ agendas to those of patients, and clinicians reported eliciting patients’ priorities and the use of theoretically-driven strategies such as goal-setting.

**Conclusions:**

To varying extents, clinicians were able to learn and implement new approaches to support patient self-management after brief training. They believed that cognitive behavioural and communication skills to facilitate self-management enhanced their practice. To optimise self-management support in routine care brief, skills-based, rheumatology-specific training needs to be developed, alongside ongoing clinical supervision. Further research should examine patients’ perspectives of care based on these approaches.

## Background

Inflammatory arthritis (IA) requires patients to make behavioural changes and psychosocial adjustments to manage its’ impact. Common challenges include fluctuations in pain, fatigue and flares of disease activity; disability, including restricted mobility and reduced participation in valued activities; emotional consequences; complex medication regimens; and decisions about when to seek help with symptoms [1].

Meeting these challenges and taking an active role in their own care is rarely a choice that patients can enact alone. An important influence is the dynamic created in the way care is provided [2]. Traditionally, healthcare teams have encouraged behaviour change and adjustment, but often through the didactic delivery of information and advice. In this model, interaction is mainly one-way communication from the expert clinician to the passive patient [3]. However, it is increasingly recognised that information alone seldom leads to behaviour change [4]; and that better health outcomes are achieved when patients collaborate in their healthcare decisions [5]. This collaboration requires the patient to acquire skills and information, and the clinician to offer them permission and support to take a more active role in their care [6]. This concept of patients as co-creators of their own health is central to self-management [7]. The process of self-management focuses on developing skills such as problem solving, decision making, appropriate use of healthcare resources, and action planning. The desired outcome is “the individual’s ability to manage the symptoms, treatment, physical and psychological consequences and lifestyle changes inherent in living with a chronic condition” [8] (p. 178).

Self-management approaches are often underpinned by the concept of self-efficacy: an individual’s belief in their ability to carry out activities to achieve a desired outcome [9,10]. Self-efficacy is a well established pathway through which psychosocial factors affect health functioning and outcomes, and is the organising focus of many self-management programmes [11]. A key way in which self-efficacy can be enhanced is through the use of cognitive-behavioural techniques. These look at patterns of behaviour, helping patients identify the links between their thoughts, feelings and behaviours, and how those might be driving or perpetuating a symptom or problem [12].

A review of over 550 research studies in chronic illness suggested that supporting patients to self-manage can have benefits for quality of life, clinical symptoms, and use of healthcare resources [13]; and can be cost-effective [14]. The drive to integrate self-management into clinical care is an important healthcare policy in the United Kingdom (UK) and the United States (US) [15,16]. However, guidelines are not provided on how to train staff to facilitate self-management; and there is a gap between policy and practice [7,17]. Therefore, a greater understanding is needed of the most effective strategies, and how they can be implemented [18]. Support for self-management is often delivered through group programmes as an adjunct to routine care rather than an integral characteristic of all consultations. Although there is evidence of the effectiveness of these programmes, they are not always widely available and uptake can be low [19,20]. In addition, long-term maintenance of self-management strategies is a challenge, as these tend to diminish over time [21]. It is therefore important to explore the feasibility of embedding techniques to facilitate self-management within rheumatology clinical teams. This would increase patients’ access to, and ongoing support from clinicians with an understanding of self-management [22].

Only a limited number of programmes deliver training for health professionals working in long-term physical health conditions, in how to facilitate self-management [13]. Little is known about the barriers and enablers to acquiring and implementing skills, and how they vary between professions, such as physicians, nurses, physiotherapists and occupational therapists. Understanding the challenges and benefits of translating self-management theory into practice is key for developing self-management training. This study explored the experiences of those few rheumatology clinicians who had undertaken brief skills training. There were two aims: to understand which aspects of their training they found helpful or unhelpful for learning, and to identify the barriers and facilitators to subsequently applying the skills in clinical practice.

## Methods

Qualitative methods were employed as the research question focuses on experiences and views. A purposive maximum variation sampling strategy was used to include participants with a range of clinical roles, and to enable the documentation of both diverse and common patterns [23]. Data were collected using semi-structured interviews, which were based on an interview schedule designed by the research team (see Interview schedule) and lasted between 40-75 min. The study was approved by the research ethics committee at the University of the West of England, Bristol (ref HLS/12/02/24).

### Interview schedule

Semi-structured interview schedule

Part A: Clinical role and brief training

•Can you tell me about your clinical role in the rheumatology team?

•Can you tell me what prompted you to do some training in self-management skills?

•Can you describe the training that you’ve done?

•What were the most helpful aspects of the course you did?

•What were the least helpful aspects of the course you did?

Part B: Putting skills into practice

•In what ways has brief skills training course altered your usual clinical practice?

•Can you describe the ways in which it is different from your approach before?

•How often, or when, do you use the skills that you learnt in your routine clinical role?

•Have you encountered difficulties or barriers to using the skills and techniques in practice?

•Have you found anything helpful for implementing the skills and techniques in practice?

•What would you recommend for subsequent self-management skills training programmes for rheumatologists?

Potential participants were approached in two ways: 4 tutors who provided self-management training sent invitation letters to rheumatology clinicians who had attended their training. Simultaneously, an advertisement about the study was placed in an electronic national newsletter for UK rheumatology professionals. Potential participants responded directly to the research team to ensure confidentiality. All participants provided written, informed consent.

Interviews were conducted by ED: 8 were face-to-face and took place in the hospital where the participant practiced; and 8 were conducted by telephone. Interviews were audio-recorded, transcribed verbatim and anonymised. A hybrid deductive/inductive analysis was conducted by ED [24,25]. First, data were examined deductively to identify content that fitted a priori codes, which would replicate, extend or refute existing theory about techniques to support self-management (Table 1). Second, data were analysed inductively to identify and organise content relating to the wider research aim of gaining insights into clinicians’ experience of training and implementation of self-management facilitation approaches. This content was coded into sub-themes which were then grouped to form broader, overarching themes. A sub-set of 6 transcripts were independently analysed inductively by SH, RG-H and patient partner RJ. Links were explored between themes, and the deductive and inductive analyses were combined into a cohesive single analysis report, evidenced by quotations (ED). There was then a meeting with all the co-authors to discuss and refine the emergent themes.

**Table 1:** Example of a priori coding in deductive phase of analysis

**Example of a priori code**	**Example of supporting data excerpt**
Socratic questioning	“…before I was trying to give them the information, ‘This is what you do, and this is what you’ve got to do”, I learnt to, ‘OK what do you think you might do about that?” *PT2*
Formulation	“…what’s really important for me obviously in this environment is the relation with the physical, thought, feeling, behaviour, so the hot cross bun type approach” *N2*
Goal-setting	“I find the goal setting very difficult. Because I think it also takes a long time, because people, when they’re invited to set goals, set completely unrealistic ones” *P1*
Double-sided reflection	“…the double-sided reflection, you know, ‘On one side you’re saying this; on the other side you’re saying that. You know, how are you going to get those two together? You don’t like the pain, you don’t want to take the medication, help me out here, what’s going on?’ so those types of things can be really powerful” *OT5*
Challenging thoughts	“…whereas before, I might have thought, ‘Oh right, they’ve got it in their head that they’re not going to change this,’ I’d try a little bit but I’d think, ‘Oh well, that’s it, they’re not making any sort of – they’re not giving me any indication they’re going to change.’ Whereas now I’ll push it more, I will be more challenging… it’s my confidence, and it’s also the course that I’ve been on, now I know, that’s an abnormal thought” *OT4*

## Results

Sixteen rheumatology clinicians (3 rheumatologists, 3 physiotherapists, 4 nurses, 6 occupational therapists), from 11 National Health Service (NHS) hospitals across the UK, took part. There were 13 women and 3 men, ranging in age from 29 to 62 years (mean 45.6), and number of years qualified from 8 to 35 (mean 20.3). Typically, training took two to three days, was led by nurses, psychologists or occupational therapists, and was generic (i.e. designed for healthcare professionals working with a variety of clinical populations). The content of training included cognitive-behavioural techniques, shared agenda setting, and goal setting (Table 2). We have used ‘approaches to facilitate self-management’ to describe the range of skills addressed in the different training programmes.

**Table 2:** Brief training programmes in approaches to facilitate self-management

Participants*	**Description of training**	**Duration**
N1; OT5;	Self-management of long term conditions	4 half days
PT1; PT2; P1		
OT1; OT2;	Training to deliver lifestyle management programme using behavioural approaches **	2 days
OT3; OT4		
N2; N3; OT6	Introduction to cognitive-behavioural therapy for long term conditions	3 days
PT3	Introduction to cognitive-behavioural therapy for long term conditions	3 days
P2	Goal-setting strategies and communications skills	10 hours
P3	Holistic care based on principles of salutogenesis (factors that support human health and well-being)	20 days***
N4	Self-management of health condition, medication and lifestyle; plus patient empowerment day	2 days

*Key: OT = occupational therapist; PT = physiotherapist; N = nurse; P = physician.

**rheumatology-specific training for occupational therapists.

***training delivered in single days over a 12 month period.

**Figure 1:**
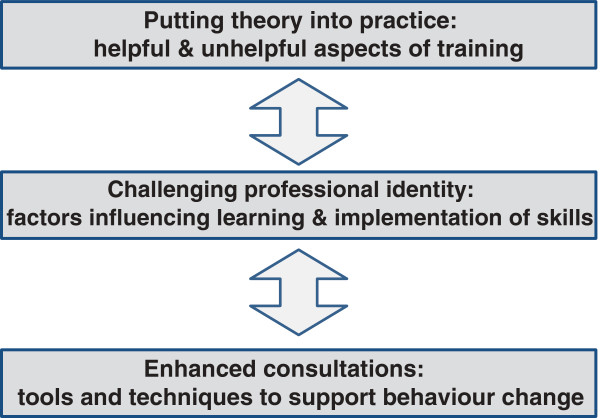
Rheumatology clinicians’ experiences of brief training and implementation of skills to support patient self-management.

Three overall themes captured rheumatology clinicians’ experiences of brief training to support patients to self-manage: Theme 1 described aspects of their training that participants had found helpful and unhelpful; Theme 2 described the impact of professional identities, working cultures and models of care on learning and implementing skills; and Theme 3 described how participants’ clinical practice had been shaped by their training programmes (Figure 1).

### Theme 1: putting theory into practice

Participants reflected on usefulness of the courses they had attended, and gave their recommendations for future self-management training.

#### Balancing theory and practice

Finding suitable courses had been difficult for some participants. Ideally, training should be brief, because time away from clinic was often an issue. It should enable clinicians to address the impact of a physical health condition within the context of their existing clinical role (e.g. disease control or function).

*“… it’s really difficult to find courses out there that offer a sort of clinician’s way in*… *it’s about building that bridge from the academic research to the clinical practice” (OT1)*

Not having information on the course content beforehand was identified as unhelpful; and led to some participants feeling out of their depth initially, and unsure about the aims and intended outcomes of their training.

“I would have liked information before each session to have read about it. ‘ Cos the first time we got there I was really like, ‘What’s going on?, it was really quite overwhelming” (N2).

Being given an explanation of the theory underpinning self-management was useful, and learning about the evidence base often strengthened participants’ confidence in the approaches. The opportunity to practice specific skills and get feedback was highly valued. The use of case studies and vignettes to exemplify the theoretical approaches to self-management helped participants to think about ways of taking these different approaches into practice.

“I think case studies are really useful. I think maybe exploring old, or like patients you’ve seen before, who you have struggled with…my example I brought was someone who would, you know, she had to clean her whole house every day. And actually that was really useful to look down about, you know, why does she feel that, and what’s the thought behind that, and what’s the belief, and then the action, and can we change any of those?” (PT2)

Participants found it helpful when tutors acknowledged some of the challenges of putting skills into practice; for example, trying to use new approaches, which were perceived as more time-consuming, with limited time to see patients. A concern experienced by many participants was in relation to using new skills competently, and how to respond if complex psychological difficulties emerged in a consultation. This scenario was perceived as more likely after brief training because participants were adopting a more holistic, exploratory approach in their interactions, during which patients might reveal deeper problems.

“…sometimes the worry about opening up these cans of worms that you then can’t manage can be quite scary initially” (PT1).

Discussions about delineating skills limitations and boundaries initially arose during training. These issues were developed further for those participants who subsequently had access to clinical supervision.

#### Access to clinical supervision

A clear sense emerged that formal training was the start of a learning process. Participants’ confidence to use and build on self-management facilitation skills in practice was highly influenced by subsequent access to clinical supervision. Regular support from someone experienced in self-management approaches was identified as necessary to overcome initial anxiety, provide encouragement, and for monitoring practice until the skills became assimilated. Those participants who were incorporating more advanced skills often used clinical supervision sessions to reflect on their boundaries in relation to the scope of their role.

“I had constant clinical supervision with **** [nurse] during my first year. And as I sort of moved from being a novice in terms of using those skills gleaned from that course, and became more confident in clinic” (N3).

By contrast, those participants who did not have access to regular and appropriate supervision found that their skills and their confidence levels could start to recede over time.

#### Support for team-wide understanding of facilitating self-management

Participants identified some disadvantages of generic training, and the potential benefits of rheumatology-specific training.

“There were people from rheumatology, some GPs from the community, physios from the community. And again some of the ideas, obviously the idea is it can be put across a broad base of disease profiles, if you like, but in some ways everyone was too diverse to converge” (PT1).

This incorporated the idea that team-wide support for self-management would enhance its implementation and effectiveness. Participants reflected that it would be beneficial if there was a team-wide familiarity with the basic theories of facilitating self-management, its clinical relevance and role in patient care. Individual clinicians would then develop their skills further and be able to focus in more depth on supporting behaviour change.

“What would have been useful was to go back with your little team, you know ‘Let’s talk about how we can implement this in our practice’, because everyone is slightly different in how they practise. So I think probably like team specific training” (PT2).

### Theme 2: challenging professional identity

The extent to which skills were implemented, and the degree of difficulty to make those changes, varied between participants. Professional identities, working cultures and models of care influenced the process.

#### A tradition of clinician-centred care

Physicians participating in the study discussed how the concept of a collaborative relationship between clinician and patient, which is the cornerstone of a self-management model of care, contrasted with a paternalistic ethos of the expert medical professional knowing what was best for the patient.

“People are very locked into the idea that they are the only ones that can truly judge for the patient what’s important for the patient, and, ‘It’s all very well to talk about patients’ symptoms and things but actually I’m the doctor and I know best’” (P1).

Several participants identified that they gained validation in their role as a healthcare professional from problem-solving on behalf of patients, which conflicted with the theory of enhancing patients’ self-efficacy. This tendency to want to ‘make things better’ was often identified during training as a way of working that could be difficult to change.

“As nurses, we like to fix people, we like to make things better; we don’t like to think that maybe what we’re doing isn’t right” (N2).

#### Taking a holistic approach to patient care

Several of the physiotherapists in the study described how their role was viewed by colleagues as being to focus primarily on physical symptoms and function.

“Physios, by nature, tend to be quite closed minded, as in focused on the problem, so the ankle, the sprained ankle or the back pain… we had a student who came to watch us working in the pain clinic, and he was like, “Well you’re just being – it’s like you’re being a psychologist.” And my colleague was like, “Well we’re not, we’re just being open-minded physios” (PT2). 

By contrast, the occupational therapists tended to be more familiar with a model of care that emphasised a holistic mind and body interaction. For those participants, training often reminded them of the mission and values attached to their profession.

“…you tend to become, or have the potential to become quite minimalist or reductionist or medically focused. So, for me, it actually reinforced that getting back to sort of proper OT, let’s really look at the patient here and not just respond to a referral to splint a wrist” (OT6).

The varied nature of the challenges and benefits, in relation to the different professions and individual experiences, led to the question of which disciplines might learn and use skills to support self-management. The most widely held view was that while some individual clinicians gravitate more naturally to working in this way, they were essentially learnable skills for the whole team.

“I do think that people can learn if they want to. But I think there’s also kind of a – you know, it’s the type of person you are, your beliefs, your profession” (OT4).

#### Impact of attitudes in the wider team

Several participants found colleagues or managers questioned the appropriateness and validity of these strategies to facilitate self-management.

“…one or two people were sort of saying, ‘Well this isn’t our problem; this is the GP’s problem if people have got emotional problems. We’re a rheumatology department.’ And one of the consultants did just say to me, ‘You’ve just got to be a little bit careful what – how you extend your role, and make sure that you are still doing your core role’” (N1).

This environment made it more difficult to access support and resources..

“When I’ve ever talked to my managers about that [clinical supervision] I’ve always been told, ‘You don’t need it because you shouldn’t be doing counselling’” (OT1).

### Theme 3: enhanced practice

Participants described how they used theoretically-driven strategies to support self-management in clinical practice, after their brief training. There was variation in the extent and range of skills employed, but all participants identified some changes which they believed enhanced their consultations.

#### A shift from the clinician agenda to the patient agenda

This was achieved using a range of questions at the start of the consultation to prompt patients to think about their priorities. Setting the agenda then became a shared endeavour; with the effect of changing the balance of the interaction, so that the patient did more of the talking and the clinician did more of the listening.

“…ask not tell, so to try and frame it as ‘What do you want to do?’…or, ‘Anything about your health you’d like to change? And I say, ‘Well it’s your appointment. We’ve got 15 min, what do you want to talk about?’ …because patients, seem to think it’s the doctor’s appointment, whereas in fact it’s a shared encounter” [P2].

By adopting this ‘Socratic’ questioning approach, participants felt that patients were more able to take responsibility for their treatment, gain a greater sense of control and problem solve. However, this move away from a clinician-led consultation was not perceived to be appropriate and acceptable to all patients and clinical practice was adapted accordingly.

*“There are some [patients]…they’re very much set in the old way, ‘Well you’re the expert, tell me what to do.’ And so you’ve got to be – pick and choose a little bit”* (*PT3).*

*Tools and techniques to support behaviour change:* Once the agreement to look at the patients’ priorities was established, participants found a range of tools and techniques helpful to identify problems and support behaviour change. Among the most widely used was formulation, sometimes known as the ‘hot cross bun’; a diagrammatic representation of the interactions between thoughts, feelings, symptoms and behaviours [22]. This tool enabled participants to work with the patient to unpick the impact that internal and external factors were having.

“…a patient will talk me through an issue or a situation or they’re a bit worried about…we’ll write down what the patient is thinking, what their mood is like …And they can then start to identify how their mood is impacting on what they’re thinking, and what they’re thinking can impact on their mood. And I’ll separate that off into behaviours, health behaviours – usually avoidance…it is a bit of a wow factor for them…So I always photocopy it and give a copy to the patient” (OT6).

Several other techniques, such as double-sided reflection, gently challenging beliefs, and interpreting pros and cons of a behaviour, were often effective for helping patients to analyse their current situation, re-think behaviours and open up possible ways forward. Goal setting was an aspect of training that several participants found difficult to incorporate into their practice, typically due long periods between visits (goal-setting requires regular review and updating or addressing problems).

“I have really struggled with the goal setting and then goal follow-up…I set a goal with a chap to do a bit of exercise and I said, ‘Right, I’ll follow up.’ Now because I wasn’t going to see him for his arthritis for about four months or something, or possibly even longer, that seemed far too long” (P2).

However, those who used this approach described getting the patient to analyse the small steps needed to reach the longer term goal; then using rating scales to check personal importance and confidence that the goal was realistic.

“…I talk about how you would actually sort of look at activity that you want to do and get back to how you can hone it down to sort of one thing, break it down into all its parts, and then all the steps that are needed to actually get you to the place you want to be. And then using the same system that **** [programme tutor] taught us about, a reward at the end, and about scoring your likelihood to achieve it.” (OT3)

## Discussion

This study explored the experiences of rheumatology clinicians who had undertaken brief training in approaches to support self-management. The aims were to understand which aspects were helpful or unhelpful, and to identify the barriers and facilitators of applying the skills in clinical practice. Three overarching themes were identified: putting theory into practice; challenging professional identity; and enhanced practice.

In Theme 1, participants identified the need for training to be brief, have an applied focus, and balance theory with time to practice skills. One issue to emerge was a preference for condition-specific training, using examples from rheumatology. This mirrors the finding from the patient perspective, that condition-specific self-management interventions are more effective than generic ones; and suggests the value of shared experience in a learning environment [26]. Participants thought that it would be beneficial if whole clinical teams could train together. This could generate a better understanding of self-management and increase the likelihood of wide adoption of these approaches [27]. However, participants acknowledged the logistical challenges of teams attending training at the same time. A key element of this theme was the positive impact of clinical supervision to help embed skills in clinical practice, and develop more advanced techniques over time. Transfer of training theory proposes that higher levels of supervision increase the likelihood of the successful transfer of learning outcomes into changes in practice [28]. This was found in research into brief cognitive-behavioural training for clinicians working in palliative care, where clinical supervision was necessary to maintain skills and build confidence [29].

Theme 2 captured how the professional identities, working cultures and models of care of the clinicians influenced learning and implementation of self-management approaches. Job involvement (the degree to which someone identifies with their professional role, actively participates in it, and considers it important to their self-worth) has been established as an important factor in implementing skills after training. High job involvement increases the motivation to transfer learning to the work setting [30]. In this current study, participants’ reflection on their professional role and their decision to undertake training to enhance their clinical skills could indicate high job involvement. Although many participants found that training made a positive difference to their clinical practice, support for using self-management approaches was not always widespread among their peers and managers. This is a significant finding, as research has found that a supportive work environment increases the likelihood that skills and knowledge acquired in training will be maintained over time [31]. It is possible that the levels of organisational and team support will change over time, as it has been recognised that clinicians need skills in self-management support to assist patients to better adhere to medical management and lifestyle behaviour change, if optimal health outcomes are to be achieved and costs are to be contained [32,33]. This theme showed that supporting self-management can be viewed as the responsibility of the team. However, clinicians in different professions are likely to work at different levels, and the important issue becomes effective use of the range of skills within the team [34]. A study with general practitioners found that training and facilitation of self-management had to be within the framework of addressing biomedical aspects of care, as this was deemed to be their primary role [35]. Therefore, it would be helpful to acknowledge the perceived remits of different rheumatology clinicians during skills training, and explore how skills to facilitate self-management can be used in their particular context. Participants’ descriptions of the impact of the wider team highlight that individual clinicians, managers, and systems are all needed to bring about change and to incorporate theoretically-informed approaches to supporting self-management into existing ways of working [27].

In Theme 3, participants perceived their consultations to be enhanced as they moved away from didactic advice-giving; talking less and listening more. This shift from clinician-dominated dialogues to interactions in which patients were helped to take a more active role fits with the UK and US promotion of a patient-centred approach to healthcare provision [36-38]. The techniques participants used, such as formulation and reflection, reinforce existing theory about the ways in which using cognitive-behavioural approaches can support self-management [22,39,40]. The data also highlighted many other techniques that some clinicians thought useful. Some techniques, such as goal setting, tended to be used more by physiotherapists and occupational therapists, where the clinical context enabled follow up within a shorter time-frame. Participants identified the creation of written or diagrammatic records during consultation, which patients then take away with them, as particularly useful. This extends our understanding of the type of material that can be helpful [41], and how it can be done through the co-production of resources by the patient and clinician. After brief training, participants were facilitating self-management to varying degrees, such as supporting patients to identify problems from their own point of view, and learn problem-solving skills which could be applied to medical, social and emotional aspects of IA [42].

Due to the low numbers of clinicians who have done brief skills training, the sample size was small for some of the disciplines. In addition, the interviews were not based on a specific course and consequently there was variation in the length and the content of the various training which each of the participants undertook. However, the focus was on understanding participants’ views of their training and impact on practice, and overall there were many commonalities in experiences. The clinicians who participated in this study were more likely to be those who were pursuing supportive self-management approaches than those who had not found training relevant or useful. Not all participants were self-management ‘naïve’ when they underwent training, and in these cases, it was a challenge to unpick which skills had been acquired during the training, rather than developed through other means.

Improving clinicians’ skills to help patients manage their own conditions has been identified as a priority for service development [43,44]. Training and implementation strategies are being developed for a number of long term conditions in primary care, for example with diabetes, chronic obstructive pulmonary disease, and irritable bowel syndrome [45,46]. However, the treatment of IA remains largely within secondary care, which offers different challenges and opportunities. Therefore, based on this study, several recommendations for brief training are proposed: provision of outline and reading material beforehand; use of practice sessions in formulation, agenda setting and goal setting; and understanding boundaries and the support available to the clinician. They should be rheumatology specific, to set practice and theory in a relevant clinical setting.

## Conclusions

A theoretical basis for supporting self-management skills and how to implement these can be learnt by rheumatology clinicians from a range of professions, after brief training. Most of those clinicians who attended such training courses found they had enhanced their clinical practice. Access to simple clinical supervision is necessary to gain confidence and embed skills. Further studies are currently investigating patients’ experiences of the impact of collaborative interaction in consultations, and rheumatology-specific training will be developed and tested.

## Competing interests

The authors declare that they have no competing interests.

## Authors’ contributions

ED was involved in the study conceptualisation and design, and data collection, analysis and interpretation. SH was involved in the study conceptualisation and design, and data analysis and interpretation. NA was involved in the study conceptualisation and data interpretation. RJ was involved in data analysis and interpretation. JC was involved in data interpretation. RG-H was involved in the study design, data analysis and interpretation. All authors read and approved the final manuscript.

## Authors’ information

ED (PhD, CPsychol) is a chartered psychologist and senior research fellow at the University of the West of England

SH (PhD, FRCN RN) is Professor of Rheumatology Nursing at the University of the West of England,

NA (PsychD) is a consultant clinical psychologist and head of the pain management team at North Bristol NHS Trust

RJ is a patient research partner in Academic Rheumatology at University Hospitals Bristol, Bristol

JC is a patient research partner in Academic Rheumatology at University Hospitals Bristol, Bristol

RG-H (PhD) is a senior research fellow and head of Health Services Research in the Musculoskeletal Research Unit at the University of Bristol

## Pre-publication history

The pre-publication history for this paper can be accessed here:

http://www.biomedcentral.com/1471-2474/15/108/prepub
